# Optimizing Foliar Spray Intervals and Rates of Isocycloseram and Cyantraniliprole Plus Thiamethoxam Application on *Hydrangea paniculata* to Combat Adult *Systena frontalis* (F) (Coleoptera: Chrysomelidae)

**DOI:** 10.3390/insects16111082

**Published:** 2025-10-23

**Authors:** Shimat V. Joseph

**Affiliations:** Department of Entomology, University of Georgia, 1109 Experiment Street, Griffin, GA 30223, USA; svjoseph@uga.edu

**Keywords:** redheaded flea beetle, *Hydrangea paniculata*, ornamental nurseries, chemical control, diamide insecticide

## Abstract

**Simple Summary:**

Redheaded flea beetle (*Systena frontalis*) has emerged as a serious pest in ornamental container nurseries in the eastern USA. Adult feeding causes economic damage to many ornamental crops, resulting in unsalable container plants. Because they undergo multiple generations in southern states, such as Georgia (USA), there is a need to manage their populations throughout the growing season, which involves multiple foliar applications to reduce adult populations and defoliation. Isocycloseram (Plinazolin^®^ Technology) is a new insecticide under consideration for registration in the USA. Cyantraniliprole + thiamethoxam (Mainspring^®^ Xtra) is a newly formulated insecticide available to growers. The objective of this study was to determine the efficacy of these insecticides on adult *S. frontalis* at various rates, and application intervals and frequencies. The results showed that isocycloseram and cyantraniliprole + thiamethoxam effectively reduced adult *S. frontalis* densities and their feeding damage compared to the nontreated control. When the adult *S. frontalis* population was high, 2–3 repeated applications of these insecticides at closer intervals reduced the feeding damage on leaves. Further research is needed to determine how these chemical tactics align with the phenology of *S. frontalis* in ornamental container nurseries.

**Abstract:**

*Systena frontalis* (Fabricius) (Coleoptera: Chrysomelidae) is a challenging pest to manage in ornamental container nurseries, affecting over 50 plant species, particularly panicled hydrangea (*Hydrangea paniculata* Siebold). Because *S. frontalis* produces multiple generations and there is a risk of developing resistance to the insecticides currently used, growers urgently seek new tools, especially new active ingredients with different modes of action or new insecticide products. Isocycloseram (Plinazolin^®^ Technology) and cyantraniliprole + thiamethoxam (Mainspring^®^ Xtra) are new potential insecticides for managing adult *S. frontalis*. However, the effective rates and application frequencies and intervals of isocycloseram and cyantraniliprole + thiamethoxam are not well understood. Therefore, the objectives of this study were to determine how rates and application intervals of these insecticides affect feeding damage when applied as foliar sprays. In laboratory assays, applying isocycloseram 1.67 SC at 59.1, 118.3, 177.4, 236.6, and 295.7 mL in 378.5 L of water, as well as cyantraniliprole + thiamethoxam at 70.9, 141.7, 212.6, and 303.3 g, reduced feeding damage compared to nontreated controls. In field trials conducted in 2024 and 2025, leaves treated with isocycloseram at 118.3 and 147.9 mL in 378.5 L of water showed significantly less damage than nontreated controls. Cyantraniliprole + thiamethoxam at 226.8 and 283.5 g in 378.5 L of water also significantly reduced the number of damaged leaves compared to nontreated controls. Two to three repeated applications of isocycloseram 1.67 SC and cyantraniliprole + thiamethoxam, administered at 7 d intervals, significantly reduced leaf damage compared to two applications at 14 d intervals.

## 1. Introduction

*Systena frontalis* (Fabricius) (Coleoptera: Chrysomelidae) is a serious pest in numerous ornamental container nurseries across eastern and Gulf regions of the USA [1,2,3,4]. *Systena frontalis* is a polyphagous pest, affecting more than 50 ornamental plant species with feeding damage that reduces crop salability [1,2,3,4]. Among these affected plants, panicled hydrangea (*Hydrangea paniculata* Siebold; Cornales: Hydrangeaceae) is particularly impacted by adult *S. frontalis* [1,2,3,4], which is a key economically important crop grown in most ornamental container nurseries across the USA. When adult *S. frontalis* feed on leaves, they remove the epidermal layer, producing shot-hole symptoms [5]. Heavy feeding and shot holes can cause the affected container plants to appear defoliated [2,4]. The larvae and pupae of *S. frontalis* develop within the growing media of plant containers, feeding on the roots (Vavilapalli, unpublished data). This feeding behaviour does not cause economic damage to nursery plants [6]. As adults emerge from the media, they mate and start feeding on leaves [6].

Adults of *S. frontalis* are small, shiny, oval-shaped insects with distinctive metallic-black bodies (~5 mm long) and bright red heads, a unique feature that sets them apart from similar beetle species [7]. The eggs are laid in the growing media and larvae and pupae reside in the same media [7]. In the first week of May, adult *S. frontalis* emerge from plant containers [6,7]. In Georgia (USA), *S. frontalis* undergoes several overlapping generations, producing adults during the growing season [4]. Adults are primarily controlled through foliar insecticide applications, which is the key tactic administered for *S. frontalis* management in container nurseries [2,4,8,9,10]. Neonicotinoids are among the most effective tools for adult *S. frontalis* in container nurseries, along with pyrethroids and diamides, especially cyclaniliprole, spinetoram + sulfoxaflor [2,11,12]. Clearly, additional active ingredients with different modes of action (Insecticide Resistance Action Committee [IRAC]) [13] are needed for season-long management of *S. frontalis* to reduce impacts on nontarget organisms and aid in managing insecticide resistance.

Isocycloseram 1.67 SC is a new insecticide being considered for registration by the United States Environmental Protection Agency for ornamental crop use in ornamental container nurseries. As an isoxazoline insecticide, isocycloseram is classified under a new IRAC group 30. Its mode of action involves acting as a gamma-aminobutyric acid (GABA)-gated chloride channel allosteric modulator [13]. Because it is a new active ingredient, it is crucial to evaluate its efficacy by understanding how different rates and application intervals affect adult *S. frontalis* and feeding damage. Similarly, cyantraniliprole + thiamethoxam (Mainspring^®^ Xtra) is a new insecticide currently available to nursery growers. It is a combination product containing cyantraniliprole and thiamethoxam, classified under IRAC Groups 28 and 4A, respectively. Cyantraniliprole targets ryanodine receptors, disrupting muscle function in insects. Thiamethoxam is a systemic, broad-spectrum insecticide within the neonicotinoid class, primarily targeting the insect nervous system through nicotinic acetylcholine receptors (nAChRs). Cyantraniliprole applied as a drench has shown effective suppression of *S. frontalis* populations [11]. Past studies did not investigate the efficacy of isocycloseram and cyantraniliprole + thiamethoxam products in detail or, specifically, the response of adult *S. frontalis* and its feeding to these products when applied at varying rates, frequencies and intervals. Therefore, this study aimed to determine the effects of varying rates, application frequencies, and intervals of isocycloseram and cyantraniliprole + thiamethoxam on the survival of adult *S. frontalis* and feeding damage when applied as a foliar spray on container panicled hydrangea.

## 2. Materials and Methods

### 2.1. Laboratory Assay, Procedure, and Evaluation

The laboratory assays were conducted in the entomology laboratory at the University of Georgia Griffin Campus, Griffin, GA, USA. For these assays, 11.4 L panicled hydrangea container plants were used, maintained in a screenhouse located on the University of Georgia Griffin Campus. The plants were irrigated for one hour daily using a sprinkler irrigation system. They were fertilized once a year with slow-release, 17-5-10 (NPK) fertilizer for up to 270 d (Florikan CRF with Gal-XeONE™; PROFILE Products LLC, Bowling Green, FL, USA). The fertilizer was top-dressed at 50 g per 11.4 L container in May 2025. *S. frontalis* adults were collected from a wholesale container nursery in McDuffie County, GA, USA. They were temporarily kept in cages (47.5 × 47.5 × 93.0 cm; BugDorm, BugDorm-4E4590 Insect Rearing Cage, Taichung 407008, Taiwan) with nontreated ‘Limelight’ panicled hydrangea container plants in a greenhouse at approximately 26 °C and 60% relative humidity. Insects were used for assays within 7 d after being introduced into the cages.

Two insecticides, isocycloseram 1.67 SC (Plinazolin^®^ Technology, Greensboro, NC, USA) and cyantraniliprole + thiamethoxam (Mainspring^®^ Xtra, Greensboro, NC, USA), were evaluated in the laboratory assay. For the isocycloseram bioassay, the six treatments consisted of (1) 0 mL, (2) 59.1 mL, (3) 118.3 mL, (4) 177.4 mL, (5) 236.6 mL, and (6) 295.7 mL of isocycloseram 1.67 SC in 378.5 L of water were tested (Table 1). There is no label available for isocycloseram 1.67 SC. This bioassay was conducted in five batches, with each batch including five replications per treatment in a completely randomized design. For the cyantraniliprole + thiamethoxam bioassay, the five treatments were (1) 0 g, (2) 70.9 g, (3) 141.7 g, (4) 212.6 g, and (5) 303.3 g of cyantraniliprole + thiamethoxam product in 378.5 L of water were tested (Table 1). Insecticide rates were selected based on their effectiveness against target insect pests, such as in Joseph 2023 [9] and their potential inclusion on future product labels. This bioassay was conducted in three batches, with each batch containing six replications in a completely randomized design. These assays were conducted using a 9 cm diameter × 1.9 cm height plastic Petri dish, as detailed in the procedure description below, with each Petri dish serving as the experimental unit.

For the assay, 2.8 cm diameter discs of panicled hydrangea leaves were prepared using a thick material puncher (Fiskars^®^; Fiskars Corporation; 2.8 cm diameter; Keilaniemi district, Espoo, Finland). Leaves that were at least two weeks old or older were used for disc preparation, with only one disc made from each leaf. In a Petri dish, a prepared leaf disc, a 5 cm diameter Whatman No. 1 filter paper, and two water-soaked cotton balls were placed before introducing the adult *S. frontalis*. The cotton balls were soaked by dipping them for 1 s in tap water. These water-soaked cotton balls provided moisture inside the Petri dish, while the filter paper absorbed any excess moisture.

The leaf discs were dipped in a specific insecticide treatment or tap water for 6 s. They were dried by placing them on a dry paper towel for one hour before introducing them into the Petri dishes. One treated or nontreated leaf disc was introduced to each Petri dish. *Systena frontalis* adults (ca. 30 adults for each batch) were collected using an aspirator from the caged panicled hydrangea maintained in the greenhouse. They were deprived of food for 12 h before being exposed to insecticide-treated or water-treated leaf discs in the Petri dishes. Five adult *S. frontalis* were introduced into each Petri dish. The leaf discs supplied food and moisture to the adult *S. frontalis* inside the Petri dish. The adult *S. frontalis* was exposed to both insecticide-treated and water-treated leaf discs for 48 h.

The leaf discs were evaluated for feeding damage at 24 h and 48 h after exposure. *Systena frontalis* feeding damage (such as shot holes and removal of the epidermal layer of tissue) on leaf discs was evaluated using a damage scale system (0–10), where 0, <1%; 1, 2–10%; 2, 11–20%; 3, 21–30%; 4, 31–40%, 5, 41–50%; 6, 51–60%; 7, 61–70%; 8, 71–80%; 9, 81–90%, and 10, 91–100% damaged area. In addition, the number of live adult *S. frontalis* was quantified at 24 h and 48 h. The live adults moved when poked with an entomological needle. Except for the first batch of isocycloseram bioassay, all assays were evaluated at 24 h and 48 h after exposure. The first batch of the isocycloseram bioassay was only evaluated for 24 h.

### 2.2. Field Site, Plant, and Insect

In 2024 and 2025, three field trials were conducted on 6527 m^2^ within a wholesale container nursery in McDuffie County, GA, USA. The three sides of the site were surrounded by more than 1000 plants, such as crape myrtle (*Lagerstroemia indica* L.), panicled hydrangea, and a woodlot on one side. The entire nursery block covers 330 ha. The plant containers around the site were infested with *S. frontalis* adults, as feeding activity on various leaves was consistently observed. A permanent sprinkler irrigation system was installed at the site, irrigating for 1 h each day unless it rained. A slow-release fertilizer (17-5-10 NPK) for up to 270 d (Florikan CRF with Gal-XeONE™; PROFILE Products LLC, Bowling Green, FL, USA) was top-dressed at 50 g per 11.4 L container once when the liners were planted in containers in September 2024.

For the trials, 11.4 L white containers of *H. paniculata* cv. ‘LimeLight’ plants were used. The growing medium for these plants was entirely coarse pine bark. They were not treated with any insecticides. The selected container plants were moved from the commercial block to the site during the last week of April 2024 and 2025. The container plants were exposed to natural populations of adult *S. frontalis* that emerged from nearby plants at the site.

### 2.3. Field Trial Design, Procedure, and Evaluation

**Figure 1:**
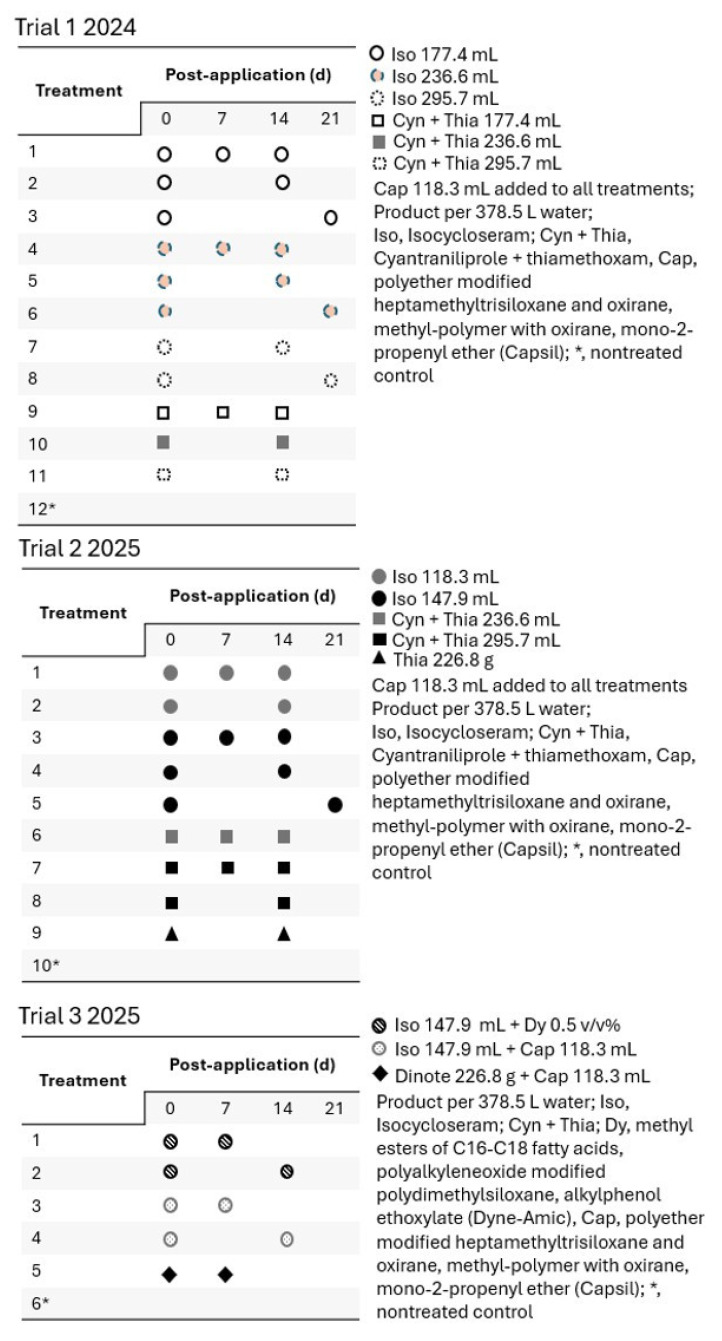
Schematic diagram of insecticides, their rates (combined with certain adjuvants) (referred to as treatments), and application intervals of treatments in trials in 2024 and 2025.

The insecticides used, their active ingredients, brand names, IRAC groups, manufacturers, and tested rates in the field trials are listed in Table 1. The treatments applied in the various trials are shown in Figure 1. The panicled hydrangea container plants were arranged in a randomized complete block design at the site. Each treatment in all the trials was replicated eight times, with an individual panicled hydrangea plant serving as the experimental unit. The treatments were blocked from the edge of the plant rows in the nursery into the trial area, assuming adults would fly into the site where trials were conducted. In April, when the plants were transferred to the site, their leaves showed no damage from *S. frontalis* feeding. The peak emergence of the first generation of adult *S. frontalis* occurred in the first or second week of May in both years. Treatments were foliar applied before adult emergence peaked, on 21 May 2024 (Trial 1) and 6 May 2025 (Trials 2 and 3). For trials 2 and 3, thiamethoxam (Flagship^®^ 25WG) and dinotefuran (Safari^®^ 20 SG) were added as industry standards.

Insecticide treatments were applied using a CO_2_-powered single-boom (one-nozzle) handheld sprayer on the panicled hydrangea container plants. The pressure was 206.8 kPa. The nozzle was connected with a flat fan nozzle (TeeJet 8002VS; TeeJet Technologies, Springfield, IL, USA). The insecticide solutions were prepared using the product rates (Table 1) in a water volume of 378.5 L. Insecticides were applied to panicled hydrangea plants at 0, 7, 14, or 21 d after treatment initiation, depending on specific treatment (Figure 1) for all three trials. After applications at designated intervals, the plants were returned to their original spots. The plant containers were spaced 0.6 m apart, both within and between blocks.

In May and June 2024, temperatures ranged from 10.6 °C to 38.3 °C, with average daily temperatures between 18.3 °C and 29.8 °C. Dew point temperatures varied from 5.6 °C to 25 °C, indicating moderate humidity levels. Rainfall was more (236.5 mm) in May compared to June (37.3 mm), with daily precipitation peaking at 75.4 mm in May and 12.4 mm in June (Thomson-McDuffie County Weather Station, Thomson, GA, USA). In May 2025, temperatures ranged from a high of 32.8 °C to a low of 8.9 °C, with average maximum, mean, and minimum temperatures of 26.9 °C, 21.4 °C, and 16.4 °C, respectively. Dew point temperatures varied between 6.1 °C and 22.8 °C, averaging 16.7 °C. No precipitation was recorded throughout the May in 2025 (Thomson-McDuffie County Weather Station).

For trial 1 (2024), the plants were evaluated at 7, 14, 21, 35, and 43 d post-first foliar application treatment, whereas in 2025 trials, they were at 7, 14, 21, 28, and 32 d post-first foliar application (Figure 1). Because adults of *S. frontalis* were yet to emerge from plant containers in the nursery at 0 d, plants were not evaluated for feeding damage. *Systena frontalis* feeding damage (such as shot holes and removal of the epidermal layer) on whole plant was evaluated using a damage scale system (0–10), where 0, <1%; 1, 2–10%; 2; 11–20%; 3, 21–30%; 4, 31–40%, 5, 41–50%; 6, 51–60%; 7, 61–70%; 8, 71–80%; 9, 81–90%, and 10, 91–100% damaged area on the plant (referred to hereafter as “damage score”) as described in Joseph (2025) [12]. In addition, the number of *S. frontalis*-damaged leaves on each plant was counted [9,12] within 1 min. A leaf with at least one noticeable feeding injury was counted as a damaged leaf.

### 2.4. Statistical Analyses

All the analyses were conducted in the Statistical Analysis System [14]. For the laboratory assays, the dependent variables, the number of adult *S. frontalis*, and the feeding damage disc score were subjected to two-way analysis of variance (ANOVA) using generalized linear mixed models (PROC GLIMMIX) with a log link function and a Poisson distribution, due to the nature of the number of adults and damage score data. The estimation method used was Laplace. The independent variables were the insecticide rate and the observation time. An interaction term between the insecticide rate and observation time was added to the model. The insecticide rate (treatment) and time of observation were the fixed effects, while replication was the random effect in the models. For the laboratory study, the data were analyzed separately for each insecticide product. Means were compared using the Tukey–Kramer test (α = 0.05). The means and standard errors were calculated using the PROC MEANS procedure in SAS (v. 9.4), with non-transformed data.

For the field trials, insecticides were applied at specific rates (referred to as L, M, and H rates) or with the addition of a type of adjuvant (Dynamic or Capsil), with treatments varying in application intervals. Not all insecticides or their rates or adjuvant addition status were applied at various application intervals (Figure 1). The dependent variables, the *S. frontalis* feeding damage score, and the number of damaged leaves were subjected to two-way ANOVA using generalized linear mixed models (PROC GLIMMIX) with a log link function and a Poisson distribution. The estimation method used was Laplace. The independent variables, insecticide and rate (plus adjuvant type), and application intervals were the fixed effects, whereas replication was the random effect in the model. An interaction variable between insecticide (rate or adjuvant type) and application intervals was added to the model. The observation time was included in the model as repeated measures. Means were separated by the Tukey–Kramer test (α = 0.05). The means and standard errors were calculated using the PROC MEANS procedure in SAS using non-transformed data.

## 3. Results

### 3.1. Laboratory Assay

**Figure 2:**
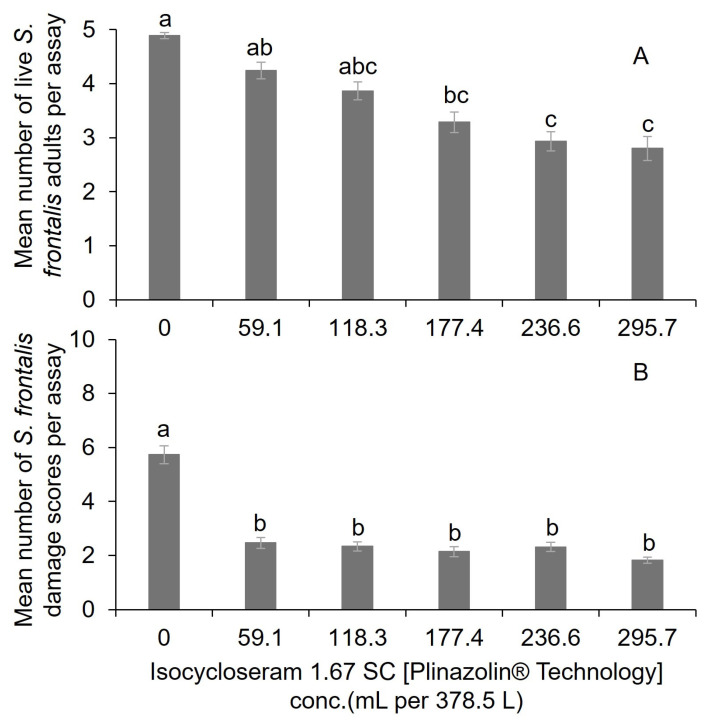
Mean (±SEM) number of (**A**) live adult *S. frontalis* and (**B**) feeding damage leaf disc scores after dipping in various rates of Isocycloseram 1.67 SC (Plinazolin^®^ Technology) solutions. The same case letter types above bars indicate no significant difference between treatments using the Tukey–Kramer test (α = 0.05).

For the isocycloseram bioassay, the mean number of live adult *S. frontalis* was significantly lower at the 236.6 and 295.7 mL per 378.5 L water treatments than at the 59.1 mL per 378.5 L water and the nontreated control (Table 2 and Figure 2A). No significant differences in mean surviving adults were observed among the 147.9, 177.4, 236.6, and 295.7 mL per 378.5 L water isocycloseram treatments (Figure 2A). The mean feeding damage scores were significantly lower for all the isocycloseram rates than for the nontreated control (Figure 2B). Except for the nontreated control, the rates of isocycloseram did not significantly differ from each other.

**Figure 3:**
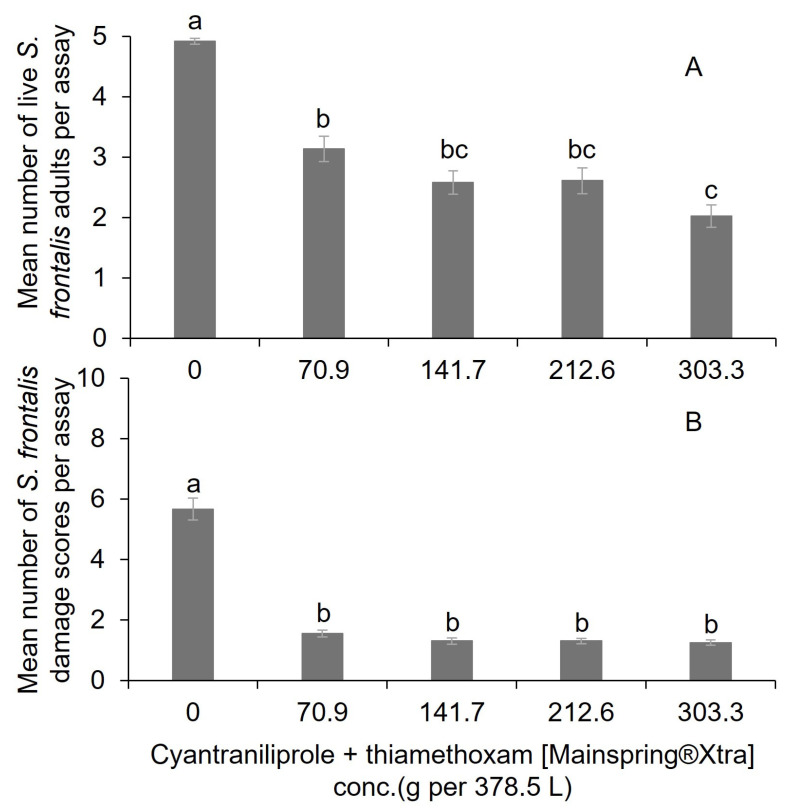
Mean (±SEM) number of (**A**) live adult *S. frontalis* and (**B**) feeding damage leaf disc scores after dipping in various rates of cyantraniliprole + thiamethoxam (Mainspring^®^ Xtra) solutions. The same case letter types above bars indicate no significant difference between treatments using the Tukey–Kramer test (α = 0.05).

For the cyantraniliprole + thiamethoxam (Mainspring Xtra^®^), significantly fewer mean live adult *S. frontalis* were observed at the 303.3 g per 378.5 L water treatment compared to the 70.9 g per 378.5 L water and the nontreated control (Table 2 and Figure 3A). Among the mean surviving adults, there were no significant differences among the 141.7, 212.6, and 303.3 g per 378.5 L water cyantraniliprole + thiamethoxam treatments (Figure 3A). The mean feeding damage scores were significantly lower for all cyantraniliprole + thiamethoxam rates compared to the nontreated control (Figure 3B). Aside from the nontreated control, the rates of cyantraniliprole + thiamethoxam did not significantly differ from each other.

### 3.2. Field Trial 1 (2024)

**Figure 4:**
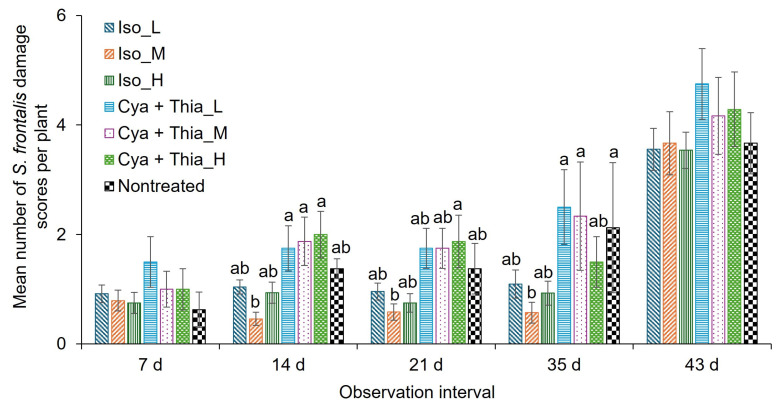
Mean (±SEM) number of damage scores after adult *S. frontalis* feeding at 7, 14, 21, 35, and 43 d post foliar spray of various rates of Isocycloseram 1.67 SC (Plinazolin^®^ Technology) and cyantraniliprole + thiamethoxam (Mainspring^®^ Xtra) at various intervals in 2024. The same case letters above bars within observation dates indicate no significant difference between treatments, as determined by the Tukey–Kramer test (α = 0.05). Where letters were not provided for certain observation dates, there were no significant differences. The abbreviations: Iso_L, 177.4 mL Isocycloseram 1.67 SC [Plinazolin^®^ Technology] per 378.5 L water; Iso_M, 236.6 mL Isocycloseram 1.67 SC [Plinazolin^®^ Technology] per 378.5 L water; Iso_H, 295.7 mL Isocycloseram 1.67 SC [Plinazolin^®^ Technology] per 378.5 L water; Cyn + Thia_L, 170.1 g cyantraniliprole + thiamethoxam [Mainspring^®^ Xtra] per 378.5 L water; Cyn + Thia_M, 226.8 g cyantraniliprole + thiamethoxam [Mainspring^®^ Xtra] per 378.5 L water; and Cyn + Thia_H, 283.5 g cyantraniliprole + thiamethoxam [Mainspring^®^ Xtra] per 378.5 L water. Capsil adjuvant 118.3 mL polyether modified heptamethyltrisiloxane (20%) and oxirane, methyl-polymer with oxirane, mono-2-propenyl ether (80%) [Capsil^®^] per 378.5 L water was added to all treatments except the nontreated treatment.

In 2024 trial 1, the insecticide treatment had a significant effect on the damage score, whereas the application interval did not (Table 3). The interaction between insecticide treatment and application interval was significantly different (Table 3). At 7 and 43 d post-first application, the insecticide treatments did not differ significantly from each other for the mean damage scores (Figure 4). At 14 d post-first application, the damage scores were significantly lower for the Iso_M treatment than for the Cya + Thia treatments (Figure 4). Between isocycloseram treatments, the mean damage scores were not significantly different from each other. At 21 d after the first application, the mean damage scores were significantly lower for the Iso_M treatment compared to the Cya + Thia_H treatment. At 35 d after the first application, the mean damage scores remained significantly lower for the Iso_M treatment than for the Cya + Thia_L, Cya + Thia_M, and the nontreated control treatments (Figure 4).

### 3.3. Field Trial 2 (2025)

**Figure 5:**
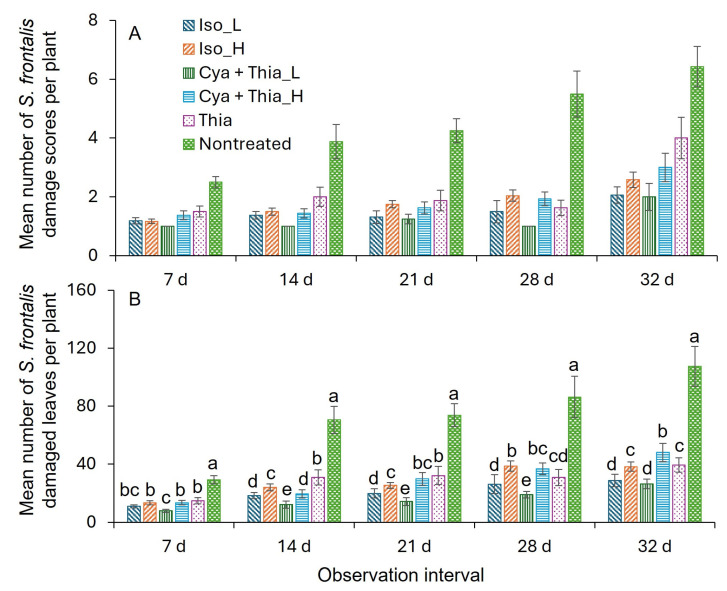
Mean (±SEM) number of (**A**) the plant feeding damage scores live adult *S. frontalis*, and (**B**) leaves damaged after adult *S. frontalis* feeding at 7, 14, 21, 28, and 32 d post foliar spray of various rates of Isocycloseram 1.67 SC (Plinazolin^®^ Technology) cyantraniliprole + thiamethoxam (Mainspring^®^ Xtra) and thiamethoxam (Flagship^®^ 25WG) in 2025 trial 2. The same case letters above bars indicate no significant difference between observation dates, as determined by the Tukey–Kramer test (α = 0.05). Where letters were not provided for certain observation dates, there were no significant differences. The abbreviations: Iso_L, 118.3 mL Isocycloseram 1.67 SC [Plinazolin^®^ Technology] per 378.5 L water; Iso_H, 147.9 mL Isocycloseram 1.67 SC [Plinazolin^®^ Technology] per 378.5 L water; Cyn + Thia_L, 226.8 g cyantraniliprole + thiamethoxam [Mainspring^®^ Xtra] per 378.5 L water; Cyn + Thia_H, 283.5 g cyantraniliprole + thiamethoxam [Mainspring^®^ Xtra] per 378.5 L water; and Thia, 226.8 g thiamethoxam [Flagship^®^ 25WG] per 378.5 L water. Adjuvant, Capsil^®^ 118.3 mL polyether-modified heptamethyltrisiloxane (20%) and oxirane, methyl-polymer with oxirane, mono-2-propenyl ether (80%) product per 378.5 L water was added to all treatments except the nontreated treatment.

There were no significant differences in the damage scores with insecticide treatment, application interval, or their interaction (Table 3 and Figure 5A). However, the insecticide treatment, application interval, and their interaction significantly influenced the leaves damaged after adult feeding (Table 3). Thus, the data on damaged leaves were analyzed in detail concerning insecticide treatment and application interval based on observation time. At all (7, 14, 21, 28, and 32 d) observation days post-first application, the mean number of leaves damaged was significantly lower for all the insecticide treatments than for the nontreated control (Figure 5B). At 7 d post-first application, the Cya + Thia_L treatment had significantly fewer mean damaged leaves than the nontreated control treatment. At 14 d after the first application, the mean number of damaged leaves was significantly lower for the Cya + Thia_L treatment compared to the Iso_L and Cya + Thia_L treatments, followed by the Iso_H treatment, then the Thia treatment, and finally the nontreated control treatment (Figure 5B). At 21 d after the first application, the mean number of damaged leaves was significantly lower in the Cya + Thia_L treatment compared to the Iso_L treatment, followed by the Iso_H treatment, then the Thia treatment, and finally the nontreated control treatment (Figure 5B). At 28 d after the first application, the mean number of damaged leaves was significantly lower for the Cya + Thia_L treatment than for the Iso_L treatment, followed by the Iso_H treatment, and then the nontreated control treatment (Figure 5B). At 32 d post-first application, the mean number of damaged leaves was significantly lower for the Cya + Thia_L and Iso_L treatments compared to the Iso_H and Thia treatments, followed by the Cya + Thia_H treatment, and then the nontreated control treatment (Figure 5B).

**Figure 6:**
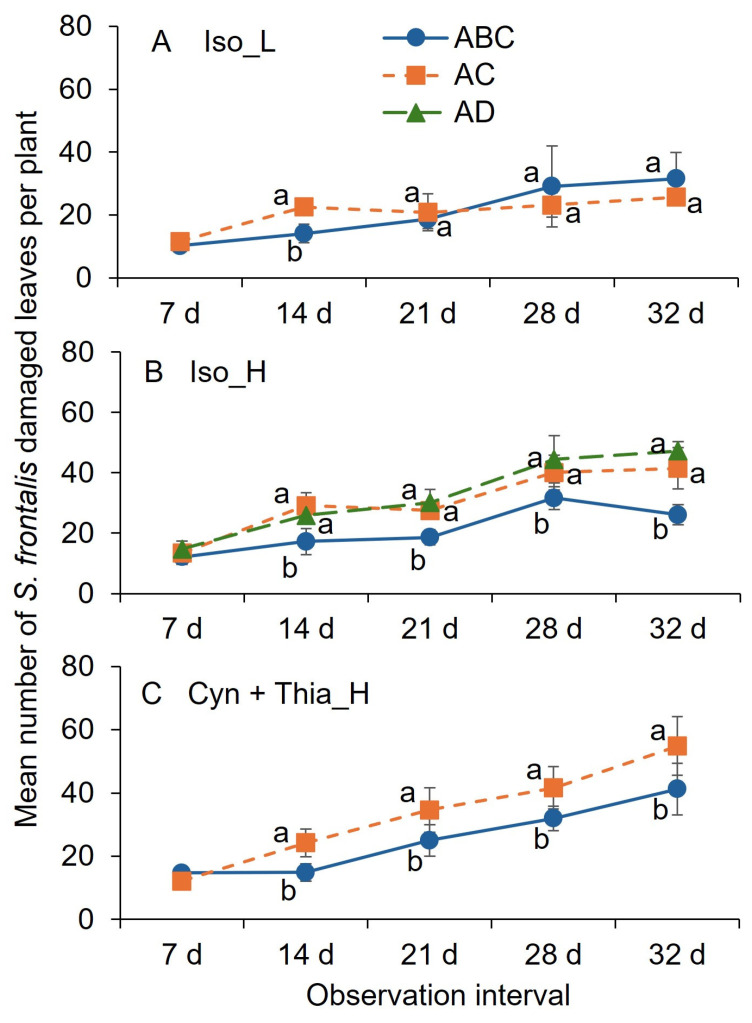
Mean (±SEM) number of leaves damaged after adult *S. frontalis* feeding at 7, 14, 21, 28, and 32 d post foliar spray of (**A**) Iso_L (118.3 mL Isocycloseram 1.67 SC [Plinazolin^®^ Technology] per 378.5 L water); (**B**) Iso_H (147.9 mL Isocycloseram 1.67 SC [Plinazolin^®^ Technology] per 378.5 L water); and (**C**) Cyn + Thia_H [283.5 g cyantraniliprole + thiamethoxam [Mainspring^®^ Xtra] per 378.5 L water) in 2025, trial 2. The same case letters above bars indicate no significant difference between observation dates, as determined by the Tukey–Kramer test (α = 0.05). Adjuvant Capsil^®^ 118.3 mL polyether modified heptamethyltrisiloxane (20%) and oxirane, methyl-polymer with oxirane, mono-2-propenyl ether (80%) [Capsil] per 378.5 L water added to all treatments except the nontreated treatment. The legend abbreviations: A, 0 d; B, 7 d; C, 14 d; and D, 21 d when the insecticide treatments were applied.

For the Iso_L treatment, at 14 d after the first application, the mean number of damaged leaves was significantly lower for the treatments applied at 0, 7, and 14 d than for the treatment applied at 0 and 14 d intervals (Figure 6A). There was no significant difference between application interval treatments of Iso_L at 7, 21, 28, and 32 d observation time. For Iso_H treatment, the mean number of damaged leaves was significantly lower for the 0, 7, and 14 d application treatments than for the 0 and 14 or 0 and 21 d interval application treatments at 14, 21, 28, and 32 d after the first application, except at 7 d after the first application (Figure 6B). There were no significant differences between 0 and 14, and 0 and 21 d application intervals of Iso_H at any observation point after the first application. For the Cya + Thia_H treatment, the mean number of damaged leaves was significantly lower for the 0, 7, and 14 d application treatments than for the 0 and 14 interval applications at 14, 21, 28, and 32 d observation time, except at 7 d observation post-first application (Figure 6C).

### 3.4. Field Trial 3 (2025)

**Figure 7:**
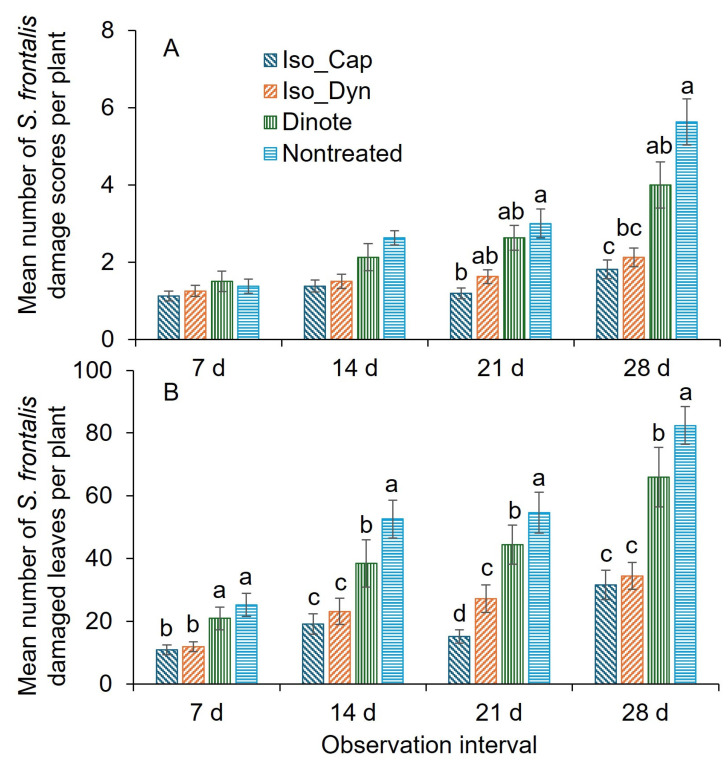
Mean (±SEM) number of (**A**) the plant feeding damage scores live adult *S. frontalis*, and (**B**) leaves damaged after adult *S. frontalis* feeding at 7, 14, 21, and 28 d post foliar spray of Isocycloseram 1.67 SC [Plinazolin^®^ Technology] and dinotefuran (Safari^®^ 20 SG) 2025 trial 3. The same case letters above bars within observation dates indicate no significant difference between treatments, as determined by the Tukey–Kramer test (α = 0.05). Where letters were not provided for certain observation dates, there were no significant differences. The abbreviations: Iso, 147.9 mL Isocycloseram 1.67 SC [Plinazolin^®^ Technology] per 378.5 L water; Dinote, 226.8 g dinotefuran product per 378.5 L water; Cap, 118.3 mL polyether modified heptamethyltrisiloxane (20%) and oxirane, methyl-polymer with oxirane, mono-2-propenyl ether (80%) product per 378.5 L water (Capsil^®^); and Dy, methyl esters of C16-C18 fatty acids, polyalkyleneoxide modified polydimethylsiloxane, alkylphenol ethoxylate (Dyn-Amic^®^, 0.5 *v*/*v*%). No adjuvant was added to the nontreated treatment.

For the damage score, the insecticide treatment and the interaction between insecticide treatment and application interval were significantly different, whereas the application interval was not significantly different (Table 3). At 7 and 14 d after the first application, there was no significant difference between insecticide treatments (Figure 7A). At 21 d post-first application, the mean damage scores were significantly lower for the Iso_Cap treatment than for the nontreated control treatment (Figure 7A). There were no significant differences in mean damage scores among Iso_Dyn, Dinote, and nontreated control groups. There were no significant differences between either Iso_Cap and Iso_Dyn treatments for the mean damage scores. At 28 d post-first application, the mean damage scores were significantly lower for the Iso_Cap and Iso_Dyn treatments than for the nontreated control treatment (Figure 7A). There were no significant differences between either Iso_Cap and Iso_Dyn treatments or Dinote and Iso_Dyn treatments for the mean damage scores (Figure 7A).

For the damaged leaves, the insecticide treatment, application interval, and interaction were significantly different (Table 3). At 7 d post-first application, the mean number of damaged leaves was significantly lower for the Iso_Cap and Iso_Dyn treatments compared to the Dinote and nontreated control treatments (Figure 7B). At 14 and 28 d post-first application, the mean number of damaged leaves was significantly lower for the Iso_Cap and Iso_Dyn treatments compared to the Dinote treatment, followed by the nontreated control treatment. At 21 d post-first application, the mean number of damaged leaves was significantly lower for the Iso_Cap treatment than for the Iso_Dyn treatment, followed by the Dinote treatment, and then the nontreated control treatment (Figure 7B).

**Figure 8:**
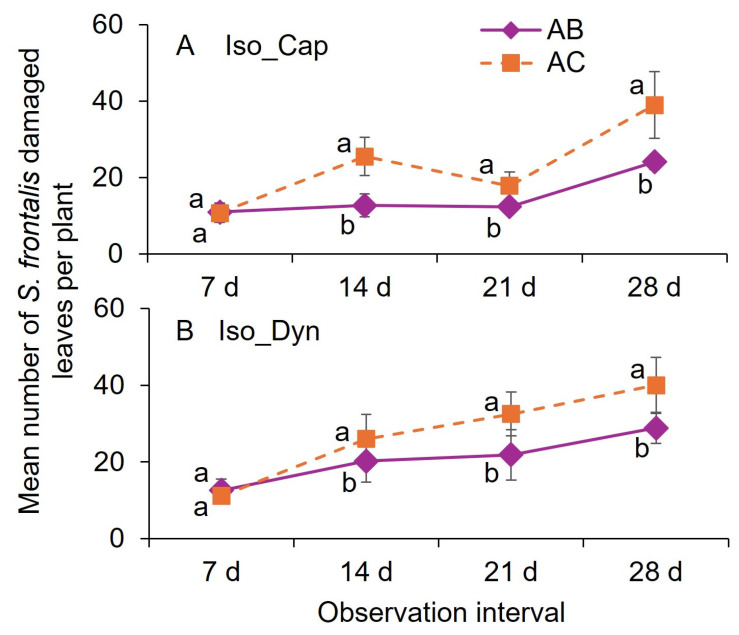
Mean (±SEM) number of leaves damaged after adult *S. frontalis* feeding at 7, 14, 21, and 28 post foliar spray of Iso, 147.9 mL Isocycloseram 1.67 SC [Plinazolin^®^ Technology] per 378.5 L water with (**A**) Cap, 118.3 mL polyether modified heptamethyltrisiloxane (20%) and oxirane, methyl-polymer with oxirane, mono-2-propenyl ether (80%) product per 378.5 L water (Capsil^®^) and (**B**) Dy, methyl esters of C16–C18 fatty acids, polyalkyleneoxide modified polydimethylsiloxane, alkylphenol ethoxylate (Dyn-Amic^®^, 0.5 *v*/*v*%) in 2025 trial 3. The same case letters above bars indicate no significant difference between observation dates using the Tukey–Kramer test (α = 0.05). The legend abbreviations: A, 0 d; B, 7 d; and C, 14 d when the insecticide treatments were applied.

For the Iso_Cap and Iso_Dyn treatments, the mean number of damaged leaves was significantly lower for the 0, and 7 d application treatments than for the 0 and 14 d interval application treatments at the 14, 21, and 28 d observation post-first application, except for the 7 d observation post-first application (Figure 8A,B).

## 4. Discussion

Results showed that isocycloseram effectively reduced adult *S. frontalis* and their feeding damage on panicled hydrangea plants, regardless of the tested rates, in both laboratory and field trials. This finding aligns with a previous study where 2–3 repeated applications of isocycloseram at 7-day intervals, using 177.4 mL and 295.7 mL in 378.5 L of water, effectively decreased the incidence and severity of feeding damage by adult *S. frontalis* for up to 29 days after the first application [9]. In the spring, the first generation of adult *S. frontalis* emerges from the growing media of plant containers, causing economic damage to the same or other container plants such as panicled hydrangeas, hollies (*Ilex* spp.), and weigela (*Weigela* spp.). Based on data, isocycloseram can protect plants from adult *S. frontalis* feeding, making it a valuable tool, especially in the spring when growers are actively selling plant containers until the end of June. For growers who do not use neonicotinoids, isocycloseram provides an alternative option for managing adult *S. frontalis* feeding damage.

In field trials, cyantraniliprole + thiamethoxam (Mainspring^®^ Xtra) yielded inconsistent results across years. Although the exact reason remains unclear, year-to-year fluctuations in population density are likely a factor. The adult *S. frontalis* population was higher in 2025 than in 2024, as indicated by the overall incidence of feeding damage across those years. Differences in insecticide treatment effects, such as leaf feeding damage, were less noticeable because adult activity was low. Nonetheless, laboratory tests demonstrated that cyantraniliprole + thiamethoxam, at a concentration as low as 70.9 g in 378.5 L of water, reduced the survival and feeding of adult *S. frontalis*. In the 2025 trial, the effectiveness of cyantraniliprole + thiamethoxam lasted up to 28 days after repeated applications when the adult *S. frontalis* population was high. Cyantraniliprole (Mainspring^®^ GNL) was moderately effective in reducing adult *S. frontalis* feeding damage in a previous study, following repeated three foliar sprays every 7 days [9]. Thiamethoxam effectively reduced adult *S. frontalis* feeding damage in this study, as well as in earlier research [15]. Therefore, the primary efficacy of cyantraniliprole + thiamethoxam appears to stem from thiamethoxam. For growers using neonicotinoids, such as imidacloprid, the granular formulation of imidacloprid can protect plants for up to five months [16], extending beyond the initial marketing period. However, those who do not use the granular formulation of imidacloprid, cyantraniliprole + thiamethoxam, or thiamethoxam alone can still guard against adult feeding. Cyantraniliprole + thiamethoxam, or thiamethoxam alone, can be applied to new plant containers, especially for panicled hydrangea, in July and August, to prepare for fall or the following year’s spring and summer sales.

When examining the application intervals, the data indicated that two or three repeated applications, spaced a week apart, performed better than applications spaced two weeks apart, regardless of whether isocycloseram or cyantraniliprole + thiamethoxam was used. This shows that fresh residues of these insecticides are crucial for providing adequate protection. The higher activity of adult *S. frontalis* in 2025 compared to 2024 was due to a larger population that year, which may have also increased the need for fresh residues on leaves to deter feeding or cause adult mortality. During the winter, the *S. frontalis* eggs hatch on days during warm spells, and the larval stages gradually develop through moulting into pupae [12]. Larval development during winter may not be synchronous due to potential factors, such as host-mediated effects, insecticide use in the previous season, or temperature variations (R. Vavilapalli unpublished data). Thus, the emergence of first-generation adults from the containers is asynchronous and extends for up to 4–5 weeks in the spring in Georgia (USA) (S. V. Joseph personal observ.). Therefore, the data from the current study suggest that repeated applications at close intervals using isocycloseram and cyantraniliprole + thiamethoxam are necessary for effective protection of adult *S. frontalis* during the spring.

Having effective insecticides in the toolbox is essential for managing *S. frontalis* throughout the season. In Georgia (USA), multiple overlapping generations occur from May to October [7]. Growers need reliable insecticides to protect their plant containers from the spring market window through subsequent market windows during the growing season. Additionally, growers regularly replenish their inventory with new plants, which also require proper protection. Having effective insecticide options extends the usefulness of isocycloseram and cyantraniliprole + thiamethoxam by reducing the need for multiple insecticide applications. This helps lower exposure to non-target organisms and prevents secondary outbreaks of minor pests, such as spider mites or scales. Since *S. frontalis* tends to re-infest plant containers and remain in the same nursery area for an extended period, repeated exposure to insecticides within the same IRAC group increases the risk of developing resistance to these insecticides. Since isocycloseram and the combination of cyantraniliprole + thiamethoxam are effective against *S. frontalis*, their use can reduce the frequency of spray applications and minimize adverse effects on non-target organisms, particularly beneficial arthropods within the system. Therefore, it is vital to have insecticides with different modes of action, and isocycloseram and cyantraniliprole + thiamethoxam would expand the options available to growers.

The current study identified two effective insecticides, isocycloseram and cyantraniliprole + thiamethoxam, for managing adult *S. frontalis* in ornamental container nurseries. When the adult *S. frontalis* population is high, these insecticides should be applied repeatedly at closer intervals to help reduce the feeding activity on plants. Although no threshold has been developed for adult *S. frontalis* feeding damage on containerized panicled hydrangea in nurseries, these insecticide products can reduce feeding damage to approximately 1%. From the current study, it is unclear whether the systemic activity of insecticides, particularly when thiamethoxam is applied as a foliar spray, can protect panicled hydrangea plants. Thus, more research is warranted to determine the length of residual activity of isocycloseram and cyantraniliprole + thiamethoxam on adult *S. frontalis* when applied to panicled hydrangea.

## Figures and Tables

**Table 1:** Insecticide products, active ingredients, and application rates used in spray trials against adult *Systena frontalis*.

Insecticide Product	Active Ingredient (%)	IRAC Group	Rate (Product Per 378.5 L Water)	Bioassay		Field Trial		
				1	2	1 (2024) ^c^	2 (2025) ^c^	3 (2025) ^d^
Plinazolin^®^ Technology	Isocycloseram 1.67 SC	30	59.1 mL	*				
			118.3 mL	*			*	
			147.9 mL				*	*
			177.4 mL	*		*		
			236.6 mL	*		*		
			295.7 mL	*		*		
Mainspring^®^ Xtra ^a^	Cyantraniliprole (20%) + thiamethoxam (20%)	28 + 4A	70.9 g		*			
			141.7 g		*			
			170.1 g			*		
			212.6 g		*			
			226.8 g			*	*	
			283.5 g			*	*	
			303.3 g		*			
Flagship^®^ 25WG ^a^	Thiamethoxam (25%)	4A	226.8 g				*	
Safari^®^ 20 SG ^b^	Dinotefuran (20%)	4A	226.8 g					*

* Rate of insecticide used in specific year; ^a^ Syngenta, Greensboro, NC, USA; ^b^ Valent Professional, San Ramon, CA, USA; ^c^ Capsil at 118.3 mL per 378.5 L water; ^d^ Dyne-Amic at 0.5 *v*/*v*% added to two Isocycloseram treatments and Capsil at 118.3 mL per 378.5 L water two Isocycloseram treatments.

**Table 2:** Statistics on live adult *S. frontalis* and feeding damage scores on panicled hydrangea discs after dipping various rates of isocycloseram and cyantraniliprole + thiamethoxam product solutions.

Insecticide	Variable	Live Beetle			Damage Score		
		*F*	df	*p*	*F*	df	*p*
Isocycloseram							
	Rate	8.9	5,231	<0.001	31.3	5,232	<0.001
	Time	16.6	1,231	<0.001	0.7	1,232	0.421
	Rate × time	1.3	5,231	0.268	1.7	5,232	0.127
Cyantraniliprole + thiamethoxam							
	Rate	14.5	4,153	<0.001	47.2	4,153	<0.001
	Time	11.7	1,153	<0.001	1.5	1,153	0.217
	Rate × time	1.1	4,153	0.353	1.4	4,153	0.251

**Table 3:** Statistics on damage scores and the number of damaged leaves from adult *S. frontalis* feeding after application of various insecticides, such as isocycloseram and cyantraniliprole + thiamethoxam products or their rates (treatment) at various intervals in 2024 and 2025.

Trial	Variable	Damage Score			Damaged Leaves		
		*F*	df	*p*	*F*	df	*p*
2024 trial 1							
	Insecticide	7.4	5,424	<0.001	-	-	-
	Application interval	1.1	2,424	0.339	-	-	-
	Insecticide × application interval	2.9	3,424	0.031	-	-	-
	Observation time	63.3	4,424	<0.001	-	-	-
2025 trial 2							
	Insecticide	1.7	4,378	0.143	44.8	4,378	<0.001
	Application interval	2.2	2,378	0.112	53.6	2,378	<0.001
	Insecticide × application interval	1.5	2,378	0.217	15.6	2,378	<0.001
	Observation time	15.2	4,378	<0.001	322.1	4,378	<0.001
2025 trial 3							
	Insecticide	8.8	2,176	<0.001	241.9	2,176	<0.001
	Application interval	1.5	1,176	0.229	82.4	1,176	<0.001
	Insecticide × application interval	0.5	1,176	0.499	4.5	1,176	0.036
	Observation time	11.4	3,176	<0.001	244.7	3,176	<0.001

## Data Availability

The raw data supporting the conclusions of this article will be made available by the authors on request.
